# Melatonin ameliorates Parkinson’s disease via regulating microglia polarization in a RORα‐dependent pathway

**DOI:** 10.1038/s41531-022-00352-5

**Published:** 2022-07-08

**Authors:** Jingwen Li, Hanshu Liu, Xinyi Wang, Yun Xia, Jinsha Huang, Tao Wang, Zhicheng Lin, Nian Xiong

**Affiliations:** 1 Department of Neurology, Union Hospital, Tongji Medical College, Huazhong University of Science and Technology, Wuhan, Hubei China; 2 Laboratory of Psychiatric Neurogenomics, McLean Hospital, Harvard Medical School, Belmont, MA USA

**Keywords:** Parkinson's disease, Neuroimmunology

## Abstract

An important pathophysiological component of Parkinson’s Disease (PD) is circadian rhythm disorder, closely related to a decrease in circulated melatonin (MLT) level. It has been reported recently that retinoic acid-associated orphan nuclear receptor (RORα), for the potentiallyendogenous ligand MLT, plays an important role in various diseases. However, the function of RORα in the pathogenesis of neurodegenerative diseases remains much unclear. Here, we showed in a cellular PD model that RORα expression was down-regulated in 1 methyl 4 phenyl pyridinium ion (MPP^+^)-treated BV2 cells but up-regulated by MLT. Of a 1-methyl-4-phenyl-1,2,3,6-tetrahydropyridine (MPTP) - induced mouse model with RORα levels reduced in the midbrain tissue, MLT treatment (intraperitoneal 20 mg/kg/d for 7 days) significantly increased the RORα levels and protected dopamine neurons, with decreased inflammation and increased anti-inflammatory M2-like phenotype in the microglia. Furthermore, siRNA-mediated knockdown implied the involvement of signal transducer and activator of transcription (STAT) pathway. In conclusion, MLT ameliorates neuroinflammation by inhibiting STAT-related pro-inflammatory (M1-like) polarization of microglia, revealing alternative options for neuroprotective treatment of PD.

## Introduction

Neuroinflammation caused by microglia activation is one of the most important mechanisms of Parkinson’s disease (PD)^1^. The death of dopaminergic (DA) neurons in the substantia nigra pars compacta (SNc) is multi-factorial and mechanisms responsible for the cell loss remain largely unknown. As the barrier of innate immunity and a main mediator of inflammatory response, microglia cells can be activated, secreting a variety of pro-inflammatory factors^2^. Microglia activation and increased inflammatory burden exacerbates the pathological consequences of PD^3^. However, the relationship between microglia activation and DA cell loss needs more investigation.

Recent evidence indicated that the detrimental role of neuroinflammation was much related to microglia status^4^. Specifically, in response to the inflammation, microglia cells develop a classic pro-inflammatory (M1-like) phenotype, releasing cytokines including interleukin-6 (IL-6), IL-1 beta (IL-1β), and tumor necrosis factor-alpha (TNF-α). In reverse, high-level inflammatory factors will strengthen local inflammatory response and further activate microglia cells again, resulting in a feed-forward loop, promoting inflammation and neurodegeneration. On the contrary, anti-inflammatory (M2-like) polarization may play a protective role, along with the release of anti-inflammatory factors such as IL-4 and IL-10^5^. Besides classic pro-inflammatory M1-like and anti-inflammatory M2-like phenotypes, another subgroup of microglial cells-Disease Associated Microglia (DAM) had been recently reported^6^. DAM microglia has both immunosuppressive and inflammatory effects in neurodegenerative disease and triggering receptor expressed on myeloid cells 2 (TREM2) was involved in this progress^7,8^. In recent publications, TREM2 has been proven to be associated with inflammatory regulation in neurodegenerative diseases^9–12^. Our previous results also highlighted the role of microglial cells in PD^13^. Therefore, it is of great urgency to understand detailed mechanisms underlying microglia polarization in PD’s neuroinflammation. The findings may provide clues for identifying new strategies to prevent DA cell loss and subsequent dyskinesia.

Melatonin (MLT) has proven effective as a treatment drug in PD patients and experiments with animal models also support that^14–16^. As an age-related hormone, MLT has been reported with broad biological effects including immunity-regulation^17^. MLT has been proved to protect tyrosine hydroxylase (TH) positive neurons (DA neurons) by reducing neuroinflammation in mouse SN_C_ and having a positive effect on the motor function of the 1-methyl-4-phenyl-1,2,3,6-tetrahydropyridine (MPTP) mouse model^18^. However, specific neuroprotective mechanisms of MLT in PD, that is, whether MLT inhibits neuroinflammation and its underlying mechanisms, remain unknown.

Retinoic acid-related orphan nuclear receptor alpha (RORα) is an important circadian nuclear receptor with a regulatory effect on immune responses. Meanwhile, RORα has recently been identified as to be the natural ligand of MLT^19^. There is growing evidence suggesting that RORα mediates multiple biological activities of MLT in various diseases including tumor and cardiovascular diseases^20,21^. In addition, previous studies have shown that the regular circadian rhythm of RORα was disturbed in Alzheimer’s Disease (AD) rat models^22,23^. However, it was still unclear how RORα was changed and regulated in PD. Disruption of the microglial clock system plays an essential role in the pathogenesis of PD^24,25^ and a recent review has proved that RORα can regulate the polarization of microglia^26^. It has been reported that the neuroprotective effects of MLT may be associated in part with its promotion of microglia M2 polarization, which would decrease the expressions of inflammatory cytokines including inducible nitric oxide synthase (iNOS)^27^. Consistently, the block of iNOS activation was correlated with the neuroprotective effect in the MPTP model of PD^28^.

At a signaling level, previous studies have found that the activation of signal transducer and activator of transcription (STAT) family played a key role in regulating macrophage/microglia polarization. It has been reported that the anti-inflammatory effect of MLT and the polarization of microglia towards M2-like phenotype were related to the increased phosphorylation of STAT3 (p-STAT3)^29,30^. By contrast, STAT1 phosphorylation (p-STAT1) is required for pro-inflammatory M1-like polarization^31,32^. However, the underlying mechanisms of polarization and inflammation regulation of microglia remain unclear in MLT against PD.

In this study, we aimed to characterize an anti-inflammatory effect of MLT in the pathogenesis of PD and RORα potential involvement in mediating the anti-inflammation.

## Results

### RORα expression was down-regulated in MPP^+^-treated BV2 cells

**Fig. 1:**
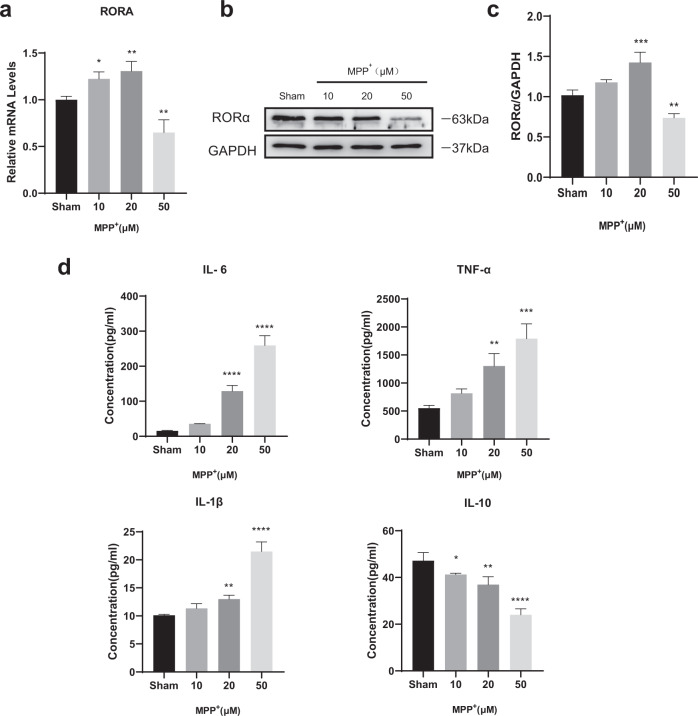
The level of RORα was reduced in BV2s treated with MPP^+^. BV2 cells were treated in different concentrations (0, 10, 20, 50 μM) of MPP^+^ for 24 h. **a** Relative mRNA levels of RORα were measured by RT-qPCR. **b**, **c** Immunoblot analysis of RORα. Protein expression levels were normalized to GAPDH. **d** ELISA assays showed the levels of IL-6, TNF-α, IL-1β, and IL-10 in culture medium. Data were average with error bars representing standard deviation. (*n* = 3 independent experiments. *P < 0.05; **P < 0.01; ***P < 0.001; ****P < 0.0001, *, vs Sham group).

To investigate whether RORα was involved in the inflammation process of PD, we first studied the biological role of RORα in BV2 cell line treated with different concentrations (0–100 μM) of 1 methyl 4 phenyl pyridinium ion (MPP^+^). Cell viability was measured and CCK-8 results showed the toxic effects. The results showed 25–30% of decrease in BV2 cell viability induced by 50 µM MPP^+^ (Supplementary Fig. 1a). Both real-time quantitative polymerase chain reaction (RT-qPCR) and western blotting (WB) analyses showed that the expressions of RORα in MPP^+^- treated BV2 cells increased at low doses of MPP^+^ (10 and 20 μM) but significantly reduced at the high dose 50 μM (Fig. 1a–c). We also observed that pro-inflammatory factors (TNF-α, IL-6, and IL-1β) gradually increased, along with the decreased anti-inflammatory factors like IL-10 as the concentration of MPP^+^ increased (Fig. 1d). The altered RORα expression combined with altered levels of cytokines suggested that RORα be an anti-inflammation player in PD.

### MLT up-regulated RORα, reduced the gene expressions of pro‐inflammation and DAM phenotype, and mitigated inflammation in MPP^+^- treated BV2 cells

**Fig. 2:**
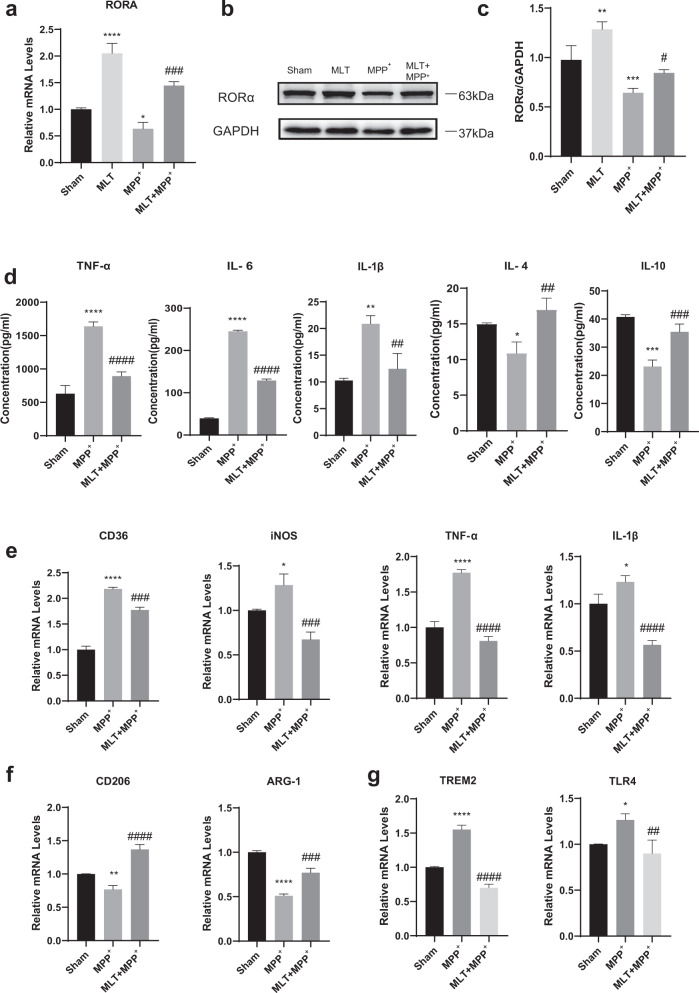
MLT increased RORα expression, inhibited pro-inflammatory M1-like microglial polarization and reduced neuroinflammation in vitro. BV2 cells were treated with MPP^+^(50 μM), MLT (50 μM) or pre-treated with MLT half an hour before MPP^+^, and then incubated for 24 h. **a** Relative mRNA levels of RORα in different groups were determined by RT-qPCR. **b**, **c** Immunoblot analysis of RORα. Protein levels were normalized to GAPDH. **d** ELISA results showed the culture medium levels of IL-6, TNF-α, IL-1β, IL-4, and IL-10. **e** mRNA quantification of pro-inflammatory (M1-like) phenotype markers (CD36, iNOS, TNF-α, IL-1β) in BV2 cells by RT-qPCR. **f** mRNA quantification of anti-inflammatory (M2-like) phenotype markers (CD206, ARG‐1) in BV2 cells. **g** mRNA quantification of DAM phenotype markers (TREM2, TLR4) in BV2 cells. Data were average with error bars representing standard deviation. (*n* = 3 independent experiments. *, #*P* < 0.05; **; ##*P* < 0.01; ***, ###*P* < 0.001; ****, ####*P* < 0.0001; *, vs Sham group; #, vs MPP^+^ group.).

The experiment of MLT treatment alone showed no harm except significant increase in RORα expression (Supplementary Fig. 1b, c), which was consistent with findings from the previous study^20^. We then explored the potential anti-inflammation mechanism for MLT on MPP^+^-treated BV2 cells. Both RT-qPCR and WB analyses showed that MLT (50 μM) completely reverse the decrease of RORα induced by MPP^+^ at 50 μM, although the final levels were lower than with MLT alone (Fig. 2a–c). Meanwhile, enzyme-linked immuno sorbent assay (ELISA) detected increased levels of anti-inflammatory cytokines IL-4 and IL-10 and significantly reduced concentrations of pro-inflammatory cytokines (IL-1β, TNF-α and IL-6) after MLT treatment (Fig. 2d), which further confirmed an anti-inflammatory effect of MLT in MPP^+^-treated BV2 cells.

Microglia polarization plays an essential role in the neuroinflammation pathogenesis during the progress of PD^33,34^. Therefore, we further analyzed the influence of MLT on microglia polarization in the cellular model. MLT was applied half an hour before MPP^+^ applied, and the markers of polarization were measured by RT-qPCR 24 h after drug intervention. The results showed that MLT treatment decreased the gene expressions for pro-inflammatory molecules such as iNOS, CD36, IL-1β, TNF-α, as well as the DAM phenotype factors such as TREM2 and Toll-like receptor (TLR) 4, but increased those for anti-inflammatory M2-like markers CD206 and arginase 1 (ARG-1) (Fig. 2e–g). These results suggested that RORα played a role in the anti-inflammatory effect of MLT, by decreasing the expressions of pro-inflammatory M1 and DAM phenotype markers in microglial cells.

### RORα expression was decreased in MPTP-treated mice and MLT showed an anti-inflammatory effect to attenuate MPTP

**Fig. 3:**
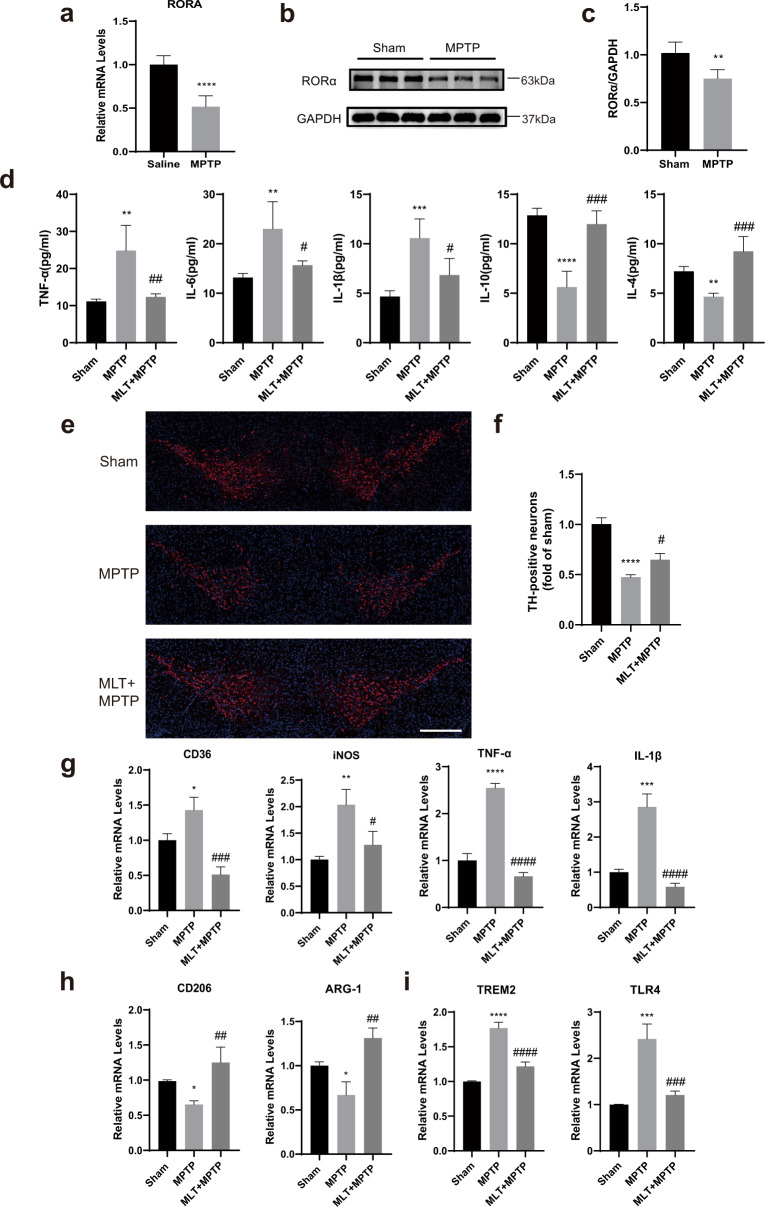
Expression of RORα was decreased in MPTP-treated mice and MLT showed an anti-inflammatory effect to attenuate MPTP. The mice were treated with MPTP (25 mg/kg, i.p.) or pre-treated with MLT (20 mg/kg, i.p.) half an hour before MPTP injection for 7 days. The levels of RORα in midbrain tissues were measured a week after the behavioural tests. **a** Relative mRNA levels of RORα were measured by RT-qPCR. **b**, **c** Protein levels of RORα, normalized to GAPDH. **d** ELISA results indicated the serum levels of TNF‐α, IL‐6, IL‐1β, IL-4 and IL‐10. **e** Representative immunofluorescent staining images of TH in SN. Blue indicates nuclei. TH were labeled as red. Scale bars = 500 μm. **f** The number of TH^+^ cells in midbrain (represented as fold of sham). **g** mRNA quantification of pro-inflammatory (M1-like) phenotype markers (CD36, iNOS, TNF-α, IL-1β) in midbrain tissue by RT-qPCR. **h** mRNA quantification of anti-inflammatory (M2-like) markers (CD206, ARG‐1) in midbrain tissue. **i** mRNA quantification of DAM phenotype markers (TREM2, TLR4) in midbrain tissue. Data were average with error bars representing standard deviation. (*n* = 5 per group. *, #*P* < 0.05; **, ##*P* < 0.01; ***, ###*P* < 0.001; ****, ####*P* < 0.0001; *, vs Sham group; #, vs MPTP group).

To investigate the relationship between RORα and PD in vivo, we used a PD mouse model induced by MPTP. Consistent with the results in vitro, RORα levels in midbrain tissue were down-regulated in MPTP-treated mice (Fig. 3a–c). To explore whether MLT had the same anti-inflammation effect in vivo, MLT was injected half an hour before MPTP injection. We observed that mice in the MLT-treated group developed better performances in motor functions compared with the control group (Supplementary Fig. 1d). The serum levels of inflammatory factors including IL-6, IL-1β, TNF-α were decreased while anti-inflammatory factor like IL-4 and IL-10 increased significantly after MLT treatment (Fig. 3d). Moreover, immunofluorescene staining showed MLT treatment could prevent the TH-positive cells from loss in the SNc (Fig. 3e, f). These results showed that MLT had anti-inflammatory and protective effects on DA neurons, thus improving the motor symptoms of MPTP-induced mouse model.

### MLT increased the levels of RORα and reduced the pro-inflammatory and DAM-related gene expressions in MPTP-treated mice

**Fig. 4:**
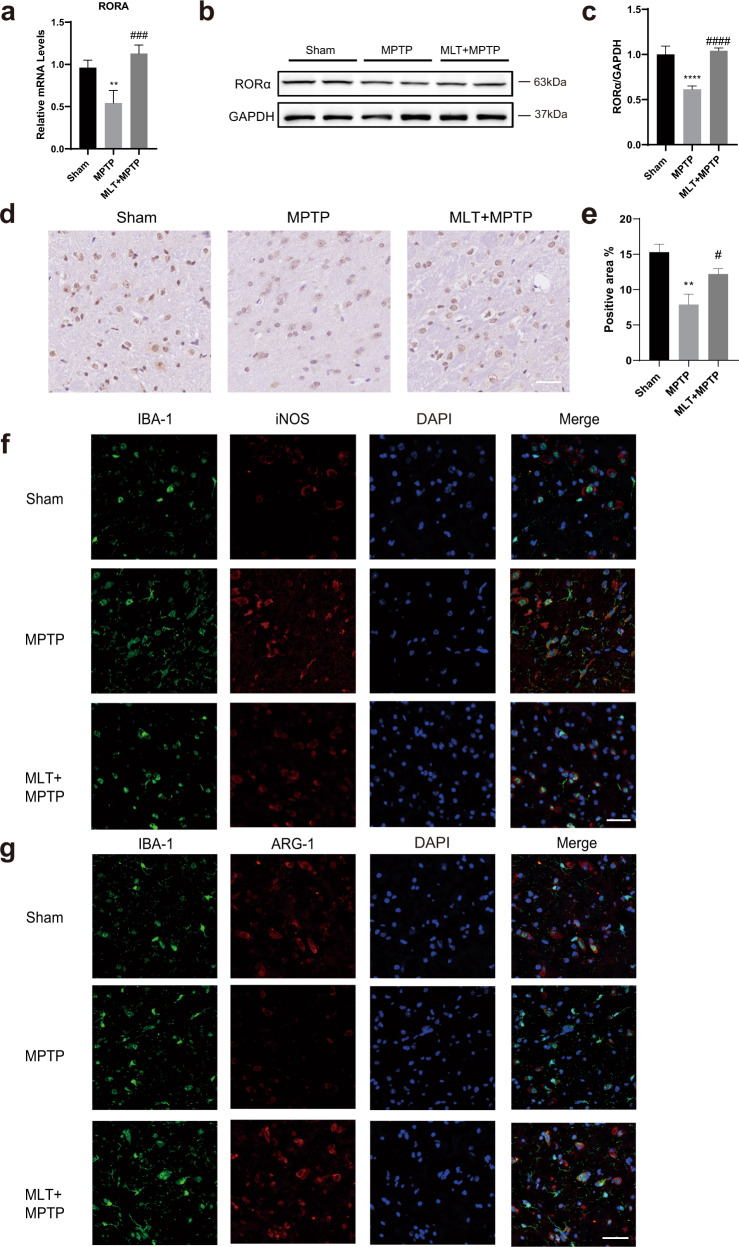
MLT treatment improved RORα expressions and directed microglia cells toward anti-inflammatory M2-like phenotype in vivo. The mice in MLT group were pre-treated with MLT (20 mg/kg, i.p.) half an hour before MPTP (25 mg/kg, i.p.) injection. MPTP, MLT or equivalent saline were applied for 7 days. **a** Relative mRNA levels of RORα in midbrain tissues were determined by RT-qPCR. **b**, **c** Protein expressions of RORα in midbrain tissue. The protein expressions were normalized to GAPDH. **d**, **e** Representative immunohistochemical images of RORα in SN. Semi-quantification of RORα protein level was presented as positive area. Scale bars = 50 μm. **f**, **g** Representative immunofluorescent staining images of IBA-1 (green), iNOS (red) and ARG-1 (red) in SN. Blue indicates nuclei. Scale bars = 50 μm. Data were average with error bars representing standard deviation. (*n* = 5 per group. *, #, *P* < 0.05; **, ##, *P* < 0.01; ***, ###, *P* < 0.001; ****, ####, *P* < 0.0001; *, vs Sham group; #, vs MPTP group).

We observed that treatment with MLT significantly increased the RORα levels in the midbrain tissue (Fig. 4a–c). Immunohistochemical staining images of RORα suggested similar results (Fig. 4d, e), suggesting that the role of MLT may be associated with RORα. Furthermore, we studied whether the microglia phenotype could be changed by MLT treatment in MPTP-induced mice. Microglia cells were activated in both MPTP and MLT + MPTP treatment groups, based on increased ionized calcium binding adapter molecule 1 (IBA-1) immunostaining. Pro-inflammatory M1-like and DAM phenotype markers reduced but anti-inflammatory M2-like phenotype molecules increased, as measured by RT-qPCR (Fig. 3g–i). Similarly, the fluorescence intensity of IBA1^+^ iNOS^+^ cells in the MLT-treatment group was less than that in the MPTP group while the fluorescence intensity of IBA1^+^ARG-1^+^ cells was higher in the MLT + MPTP group than the MPTP group (Fig. 4f, g). To sum up, MLT treatment changed the microglia phenotype via down-regulating the pro-inflammatory and DAM-related molecules.

### RORα deficiency promoted microglia pro-inflammatory phenotype polarization to aggravate neuroinflammation

**Fig. 5:**
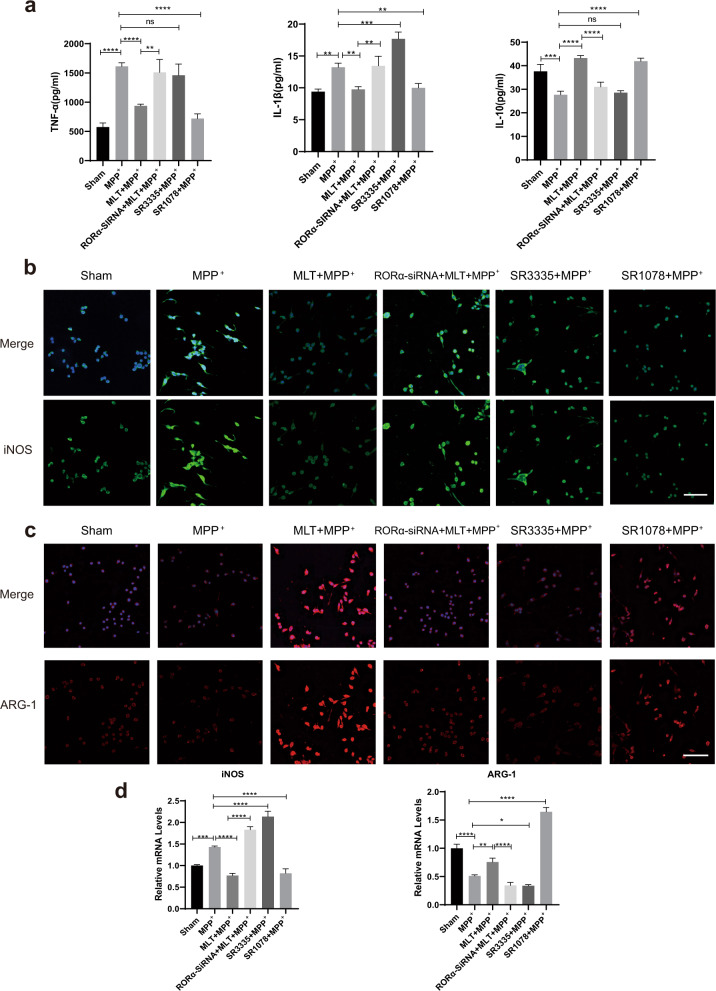
The deficiency of RORα promoted inflammation. BV2 cells were firstly transfected with siRNA for 48 h to knock down RORα expression. MPP^+^(50 μM), MLT (50 μM), SR3335 (a synthetic selective inverse agonist of RORα, 10 μM), SR1078 (a synthetic agonist of RORα, 10 μM) were applied alone or combined (MLT, SR3335 and SR1078 were treated half an hour before MPP^+^) for 24 h. **a** ELISA results indicated cell culture medium levels of TNF‐α, IL‐1β and IL‐10. **b**, **c** Immunofluorescent staining images of iNOS (green) and ARG-1 (red). Blue indicates nuclei. Scale bars = 50 μm. **d** RT-qPCR results showed the quantitative analysis of polarization markers iNOS and ARG-1 expressions in different cell groups. Data were average with error bars representing standard deviation. (*n* = 3 independent experiments.*P < 0.05; **P < .01; ***P < 0.001; ****P < 0.0001).

It has been reported that RORα could play an anti-inflammatory role in a variety of diseases^35–38^. To verify whether RORα played a similar role for the effect of MLT on MPP^+^-treated BV2 cells, we further determined the molecular effects of RORα deficiency on microglia polarization and inflammation. SiRNA was used to knock down RORα. In addition, we also used SR3335, a synthetic selective inverse agonist of RORα and SR1078, a synthetic agonist of RORα, before MPP^+^ intervention. At a protein level, both the MLT and SR1078-treated group showed anti-inflammation effects, compared with MPP^+^-treated group. However, SR3335 did not increase the level of TNF-α or decrease the level of IL-10 in MPP^+^-treated cells. More importantly, compared with the non-silence group of MLT + MPP^+^, RORα knockdown increased levels of pro-inflammatory factors (TNF-α and IL-1β) and reduced the anti-inflammatory one (IL-10) in the culture medium (Fig. 5a). Meanwhile, the up-regulation of iNOS and down-regulation of ARG-1 showed by immunofluorescence images also revealed that pro-inflammatory polarization could be augmented by RORα deficiency in BV2 cells (Fig. 5b, c). At the mRNA level, RT-qPCR showed consistent results except the ARG-1 expression in MLT-treated cells group. MLT + MPP^+^-treated cells show increased immunostaining intensity but decreased mRNA of ARG-1 (Fig. 5d). To further investigate the in vivo role of RORα on microglia polarization and inflammation, SR3335 and SR1078 were injected half an hour before MPTP injection. Meanwhile, the serum levels of pro-inflammatory factors TNF-α and IL-1β increased while IL-10 levels decreased after SR3335 treatment (Supplementary Fig. 1e). RT-qPCR results in midbrain tissue showed that SR3335 significantly increased the gene expression of pro-inflammatory marker iNOS and reduced the expression of anti-inflammatory marker ARG-1 (Supplementary Fig. 1f). The group of SR1078 showed the opposite effect (Supplementary Fig. 1e, f). These results demonstrated that RORα was involved in the anti-inflammation effect of MLT. Specifically, RORα mediated the microglia polarization to anti-inflammatory phenotype and alleviated inflammation.

### MLT regulated microglia polarization through the RORα‐dependent signaling pathway of STAT

**Fig. 6:**
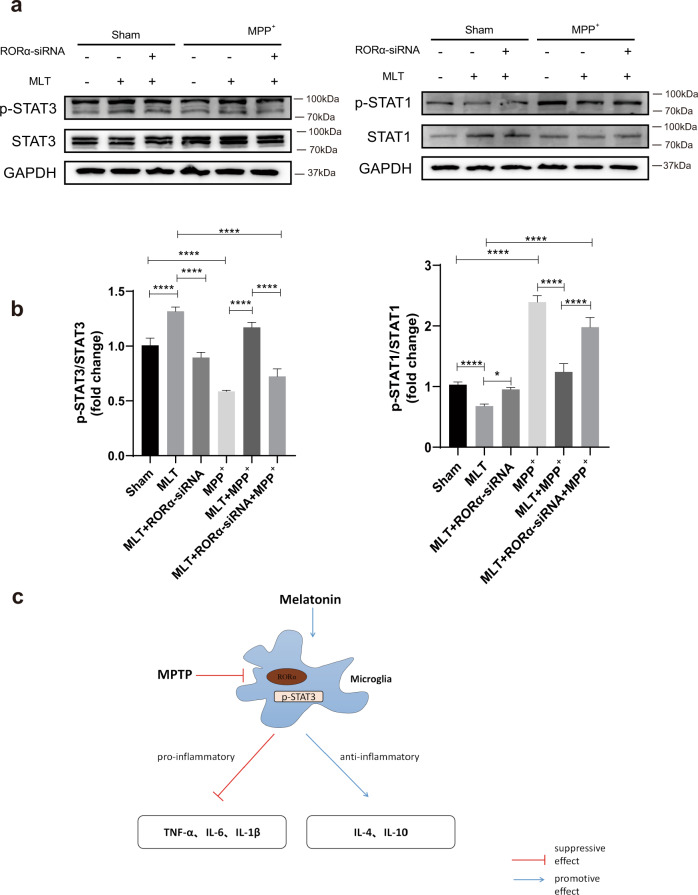
MLT regulated the phenotypic change in microglia via RORα-STAT pathway. **a**, **b** Immunoblot analysis of STAT1, STAT3, phosphorylated‐STAT1 and phosphorylated‐STAT3 in BV2 cells with MPP^+^, MLT and RORα-siRNA treatment alone or in combination for 24 h with GAPDH as a loading control. Fold change was calculated by *Image J*. Data were average with error bars representing standard deviation. (*n* = 3 independent experiments. *P < 0.05; ****P < 0.0001). **c** Assumed mechanism for the anti-inflammatory effect of melatonin via RORα against PD.

It has been reported that the anti-inflammatory effect of MLT was related to the activation of the STAT pathway^39^. To further explore the signaling pathway related to MPP^+^-induced neuroinflammation and MLT-mediated anti-inflammation, we examined the expression and phosphorylation of the key signaling molecules STAT, especially STAT1 and STAT3. Results showed that the expression of p-STAT1 was increased while p-STAT3 was significantly decreased in MPP^+^-stimulated BV2s. The pre-treatment with MLT suppressed the expression of p-STAT1 but activated STAT3 phosphorylation. In order to verify whether RORα was involved in the effects of MLT on STAT pathways, RNA interfering (siRNA) was used to knock down RORα. In comparison to the cells group without RORα-siRNA, the deficiency of RORα significantly decreased the level of p-STAT3 but increased the phosphorylation of STAT1 caused by MLT pre-treatment (Fig. 6a, b). These observations suggested that MLT mediated the phenotypic change in microglia via the RORα-STATs pathway.

## Discussion

In this research, we studied the regulatory effect of RORα in MPP^+^-activated microglia cells and in MPTP-treated mice. At low doses of MPP^+^, the increase in RORα expression was largely due to compensatory response, maintaining protective effects. When the concentration of MPP^+^ kept escalating, BV-2 cells lose their compensatory capacity and thus RORα gets down-regulated. We further demonstrated the therapeutic potential of MLT in PD. We clearly observed that MLT significantly improved the motor functions and prevented DA neurons from loss. In addition, MLT ameliorated neuroinflammation by increasing the expressions of anti-inflammatory M2-like phenotypes in microglial cells. Further investigation in BV2 cells confirmed that RORα mediated the anti‐inflammatory effect of MLT. Moreover, this MLT‐RORα axis changed the phenotype of microglia via the STATs pathway. Taken together, our study introduced an essential nuclear receptor, RORα, which was an important endogenous mediator of MLT‐exerted anti-inflammation against PD (Fig. 6c).

Here, we provided new anti-inflammation evidence of MLT in PD. MLT has been widely used in a large number of clinical and animal studies, and also been proved to have various biological benefits in PD^40^. Growing evidence demonstrated the neuroprotective effects of MLT in neurodegenerative disorders including PD^41^. Preventing inflammation and protecting DA neurons are main objectives of neurological researches. Previous researches have discussed the anti-inflammation and neuroprotective effect and the regulation of TH expression by MLT activating MT1 receptors in PD^42–44^ while MT2 receptors were reported more related to the depression in PD^45,46^. Besides, our study provides an insight into an anti-inflammatory and microglia-polarization mechanism of MLT via the nuclear receptor RORα during PD pathogenesis.

MLT‐RORα axis may be an important endogenous signaling pathway involved in regulating various pathophysiological processes including neurodegenerative diseases. As reported, RORα is involved in many important biological functions including cell metabolism, cell growth and death, immune responses and tumorigenesis. Emerging evidence suggests that RORα plays a key role in maintaining neural homeostasis. RORα deficiency may lead to a variety of diseases such as multiple sclerosis^47^, cerebral ischemia-reperfusion injury^48^, autism^49^ or cerebellar ataxia^50^,epilepsy^51^. RORα has been recently identified as a novel MLT receptor and there are many essential roles between MLT and RORα, such as circadian rhythmic and oxidative stress regulation^52^. Although the role of RORα as a MLT receptor remains some controversy^53^, the RORα‐dependent function of MLT has been convincingly proved by several studies^54–56^_._ The action of MLT might be through up-regulation of sirtuin-1, and PGC-1α^57^. Additionally, an alternative explanation is that activation of the MLT receptor may increase the RORα expression via the AKT actions^58^.

Microglia, the major immune cells in the central nervous system, play important roles at all stages of PD^59,60^. Microglia activation induces an intense inflammatory response, releasing pro-inflammatory cytokines, which initiates deterioration process. The status of microglia contributes to the neuroinflammation, which will further lead to the loss of DA neurons^61^. In our research, we have found that MLT significantly up-regulates RORα and directs microglia cells towards an anti‐inflammatory M2-phenotype. For the disagreement between the mRNA level and immunostaining intensity of ARG-1 in MLT-treated group, an explanation may be that MLT did not affect the gene activity but did enhance the translation of ARG-1 in BV2 cells. Further evidence has shown that RORα plays an anti-inflammatory role by mediating the beneficial effects of MLT.

Our results demonstrated that the MLT-regulated microglia polarization is mediated by RORα in a STAT pathway-dependent manner. Specifically, MLT inhibited STAT1 but promoted STAT3 phosphorylation in microglia cells. As reported, STAT-related pathway participates in the regulation of cytokine signaling and macrophage/microglia polarization^62^. Min KJ et al. reported that the expressions of p-STAT1 in BV2s was reduced by the intervention of MLT^63^. Another study demonstrated that MLT up-regulated p-STAT3 and thereby directed microglial cells into anti-inflammatory phenotypes^29^ while STAT1 directed microglia towards pro-inflammatory M1-like phenotype^64^. Therefore, it suggested that MLT may regulate microglia polarization through the RORα‐STATs pathway during the progress of PD.

In conclusion, this study proved that MLT alleviated neuroinflammation by changing the phenotype markers of microglia. MLT regulated microglia’s shift towards an anti-inflammation phenotype and RORα exerts a key role in the regulation of microglial polarization. This finding points to the beneficial effects of MLT‐RORα axis which may reduce inflammatory responses and ameliorate PD.

## Methods

### Animal models

A total of 52 male C57/B6J mice (8–10 weeks, 25–27 g) were subjected to regular illumination for 12 h/12 h. They were equally divided into several groups and treated with saline, MPTP (25 mg/kg, Sigma, M0896), MLT (20 mg/kg, Sigma, M5250), SR3335 (15 mg/kg, MCE, HY-14413) and SR1078 (10 mg/kg, MCE, HY-14422) alone or in combination. MLT, SR3335, or SR1078 was injected intraperitoneally (i.p.) half an hour before MPTP injection, and the duration of drug administration were 7 consecutive days^65,66^. Within a week after the behaviour tests, we dissected the midbrain tissue for subsequent analyses. This study had been approved by the Animal Ethics Committee of Tongji Medical College, Huazhong University of Science and Technology (Ethics number 2021S2576).

### Behaviour tests

Behaviour tests started one day after the final drug injection, with adaptive training, three days before the formal tests.

#### Pole test

An iron rod with a length of 50 cm and a diameter of 1 cm was placed vertically on the ground and wrapped with gauze to prevent the mouse from slipping. A mouse was put with head up on the top of the pole. Recording the time started when the mouse turned head down and tail up and stopped when both hind legs reached the bottom of the pole. In short, recorded was the time for the mouse to complete the walk-down from the top to the bottom of full-length pole^67^. An average time of five consecutive tests was estimated for each animal.

#### Rotarod test

A mouse was placed on the rotating axis of the rotary rod meter with the diameter of 3 cm, and the rotation speed was set to 20 r/min. Recorded was the duration time of the mouse on the rotating axis before falling^68^. Each mouse was tested three times and the mean value was taken, with an interval of at least one hour.

### BV2 cell culture, drug treatment and siRNA transfection

Mouse microglia cell line BV-2 was obtained from Procell Life Science & Technology Co., Ltd. (Wuhan, China). The cells were cultured in dulbecco’s modified eagle medium (DMEM, Gbico) supplemented with 10% Fetal bovine serum (FBS, Biological Industries) and 1% penicillin/streptomycin (P/S, Servicebio) at 37 °C in a 5% CO_2_ incubator. Culture medium changed once every two days. In order to explore the varying expression of RORα in vitro, BV2 cells were seeded into 6-well plates at the density of 5 × 10^5^/ml and treated with different concentrations of MPP^+^ (0,10,20,50 μM, Sigma, D048) for a duration of 24 h. In order to verify the neuroprotective effect, MLT (50 μM), SR3335 (10 μM), SR1078 (5 μM) was used for pre-treatment half an hour before MPP^+^ (50 μM) intervention. Constant volume of phosphatic buffer solution (PBS) or DMSO was used as control. SiRNA-mediated knockdown of RORα was conducted using Lipofectamine 3000 transfection agent (Invitrogen) according to the manufacturer’s protocol. All the samples were collected 24 h after MPP^+^ treatment for following experiments.

### Cell viability and CCK-8 assays

The cell viability was measured by CCK-8 (Seven Biotech) assay^69^. In brief, BV2 cells were plated in 96-well plates and treated with MPP^+^/MLT in different concentrations. After incubation of 24 h, add CCK-8 (10 μl per well) and continue incubated for 2 h at 37 °C. The absorbance was determined by measuring at 450 nm.

### Immunohistochemistry/Immunofluorescence

After perfusion, mice were decapitated and the midbrain tissues were dissected and kept in paraffin and sliced into 5μm sections. For immunohistochemistry, briefly, sections were deparaffinized and rehydrated, and heat-induced antigen retrieval was performed using sodium citrate solution (Sinopharm, 10019418), pH 6.0, for 30 min. Next, endogenous peroxidase was blocked with 3% H_2_O_2_ (Sinopharm, 10011218) for 20 min. Nonspecific binding sites were minimized with 10% goat serum (Boster, AR1009) for 20 min followed by incubation of primary antibodies against RORα (Novus, NBP1–52813, 1:100) at 4 °C overnight. On the second day, sections were washed in TBST for 10 min and then incubated with the appropriate biotinylated secondary antibody (Abcam, ab205718, 1:2000) at room temperature (RT) for one hour. Using diaminobenzine (DAB) as the chromogen (Maxim, DAB4033), sections were counterstained with hematoxylin (Baso, BA4041). All DAB-immunostained sections were mounted, dried and imaged with a slice scanner (Hamamatsu, NanoZoomer S360). Semi-quantitative analysis of RORα protein levels was presented by positive area (shown as the percentage of RORα-positive area in the SNc). Sections containing consistent SNc region/TH^+^ cells (20 sections per animal) were selected in different groups and analyzed within a uniform detection threshold using the *Image J*.

For immunofluorescence, the BV2 cells were fixed with 4% paraformaldehyde (Servicebio, Wuhan, China) for 10 min. Cells and brain sections on glass slides were both permeabilized with 0.1% Triton X-100 (Beyotime, Shanghai, China) for 20 min, blocked with 5% bovine serum albumin (Beyotime, Shanghai, China) for one hour. Paraffin sections and cells were incubated with primary antibodies including rabbit anti-TH (Proteintech, 25859-1-AP, 1:800), goat anti-IBA1 (Abcam, ab5076,1:100), rabbit anti-iNOS (Proteintech, 18985-1-AP, 1:100) and mouse anti-Arginase1 (Proteintech, 66129-1-Ig,1:100) at 4 °C overnight. They were reacted with alexa Fluor 488 or 594-conjugated corresponding secondary antibodies (Invitrogen, 1:200). And nuclei were visualized by DAPI (Solarbio, C0060). All immunostained sections were entirely scanned and fluorescence signals were imaged with a fluorescence microscope (Olympus BX53). The number of TH^+^ neurons were counted in every 30 μm through the entire midbrain from anterior to posterior^70^. For each group of brains, the mean number of TH^+^ neurons were calculated and represented as a fold change from the sham group in this study.

### Enzyme-linked immuno sorbent assay (ELISA)

After the behavioural tests, mice were sacrificed with deep anesthesia and blood was collected. The cells’ supernatant was collected after drug intervention for further assays. Serum and culture medium supernatant were collected by centrifugation at 2000 × *g* and 4 °C for 20 min and stored at −20 or −80 °C. The levels of TNF-α, IL-1β, IL-6, IL-4 and IL-10 were measured using corresponding mouse ELISA kits (Boster) following the manufacturers’ protocol.

### Western blotting (WB)

A total of 30 μg protein pool was resolved by 10% sodium dodecyl sulfate polyacrylamide gel electrophoresis (SDS-PAGE) and subsequently electro-transferred to polyvinylidene fluoride membranes (Millipore). The membrane was soaked at RT for one to two hours, followed by overnight incubation at 4 °C with the following primary antibodies (1:1000 dilution): rabbit anti-RORα polyclonal antibody (Abclonal, A6971), rabbit anti-STAT1 monoclonal antibody (Cell Signaling Technology, #14994), rabbit phospho-STAT1 monoclonal antibody (Cell Signaling Technology, #7649), mouse anti-STAT3 monoclonal antibody (Cell Signaling Technology, #9193), or rabbit antibody monoclonal phospho-STAT3 (Cell Signaling Technology, #9145). After washed in TBS-T three times for 5 min each, corresponding anti-rabbit/mouse HRP-conjugated secondary antibodies (Proteintech, SA00001-1/2, 1:10000) were added, with incubation at RT for one hour. The immunoblot bands were detected via enhanced chemiluminescence (ECL) western blot detection kit (Share-bio Biotechnology, Shanghai, China). The signal intensities were analyzed by *Image J* software. All blots derive from the same experiment and were processed in parallel.

### Real-time quantitative Polymerase Chain Reaction (RT-qPCR)

Total RNA was isolated from midbrain tissues and BV2 cells using TRIzol (Vazyme, China)^71^. cDNA was synthesized from the RNA using RT SuperMix for qPCR (Vazyme) and then amplified by SYBR qPCR master mix (Vazyme) in the ABI StepOnePlus Real-Time PCR System (Applied Biosystems, USA). The primer sequences utilized for real-time PCR are presented in Supplementary Table 1. Results from ABI StepOnePlus analysis software are quantified as Ct values, normalized to the reference gene GAPDH, and shown as 2^−∆∆Ct^.

### Statistical analysis

Results were presented as the mean ± standard deviation (SD) of measures repeated at least three times independently. All data were analyzed using *Graphpad Prism 7*. Comparison of different groups was performed by one-way ANOVA analysis, followed by Tukey’s post-hoc tests for multiple comparisons. *P*-value ≤ 0.05 was considered statistically significant.

## Supplementary information

supplementary materials

## Data Availability

All relevant data generated during this study are available from the corresponding author on reasonable request.
