# Supplementary material for: Melatonin ameliorates Parkinson’s disease via regulating microglia polarization in a RORα‐dependent pathway
Source: NPJ Parkinsons Dis. 2022 Jul 8;8:90. doi: 10.1038/s41531-022-00352-5 (PMC9270337; doi:10.1038/s41531-022-00352-5)
Supplement: Supplementary file 1 — supplementary materials [file 41531_2022_352_MOESM1_ESM.pdf]

Supplementary Table 1. Primers used in this experiment

Gene	5' Forward	3' Reverse
GAPDH	AGGTCGGTGTGAACGGATTTG	TGTAGACCATGTAGTTGAGGTCA
RORA	GTGGAGACAAATCGTCAGGAAT	TGGTCCGATCAATCAAACAGTTC
CD36	AGATGACGTGGCAAAGAACAG	CCTTGGCTAGATAACGAACTCTG
CD206	CTCTGTTCAGCTATTGGACGC	TGGCACTCCCAAACATAATTTGA
iNOS	GTTCTCAGCCCAACAATACAAGA	GTGGACGGGTCGATGTCAC
ARG-1	CTCCAAGCCAAAGTCCTTAGAG	AGGAGCTGTCATTAGGGACATC
TNF-α	CACGCTCTTCTGTCTACTGAACTTC	ATGATCTGAGTGTGAGGGTCTGG
IL-1β	GCACTACAGGCTCCGAGATGAA	GTCGTTGCTTGGTTCTCCTTGT
IL-10	AGCCTTATCGGAAATGATCCAGT	GGCCTTGTAGACACCTTGGT
TREM2	GACCTCTCCACCAGTTTCTCC	TACATGACACCCTCAAGGACTG
TLR4	CTCACAACTTCAGTGGCTGGATTT	GTCTCCACAGCCACCAGATTCTC

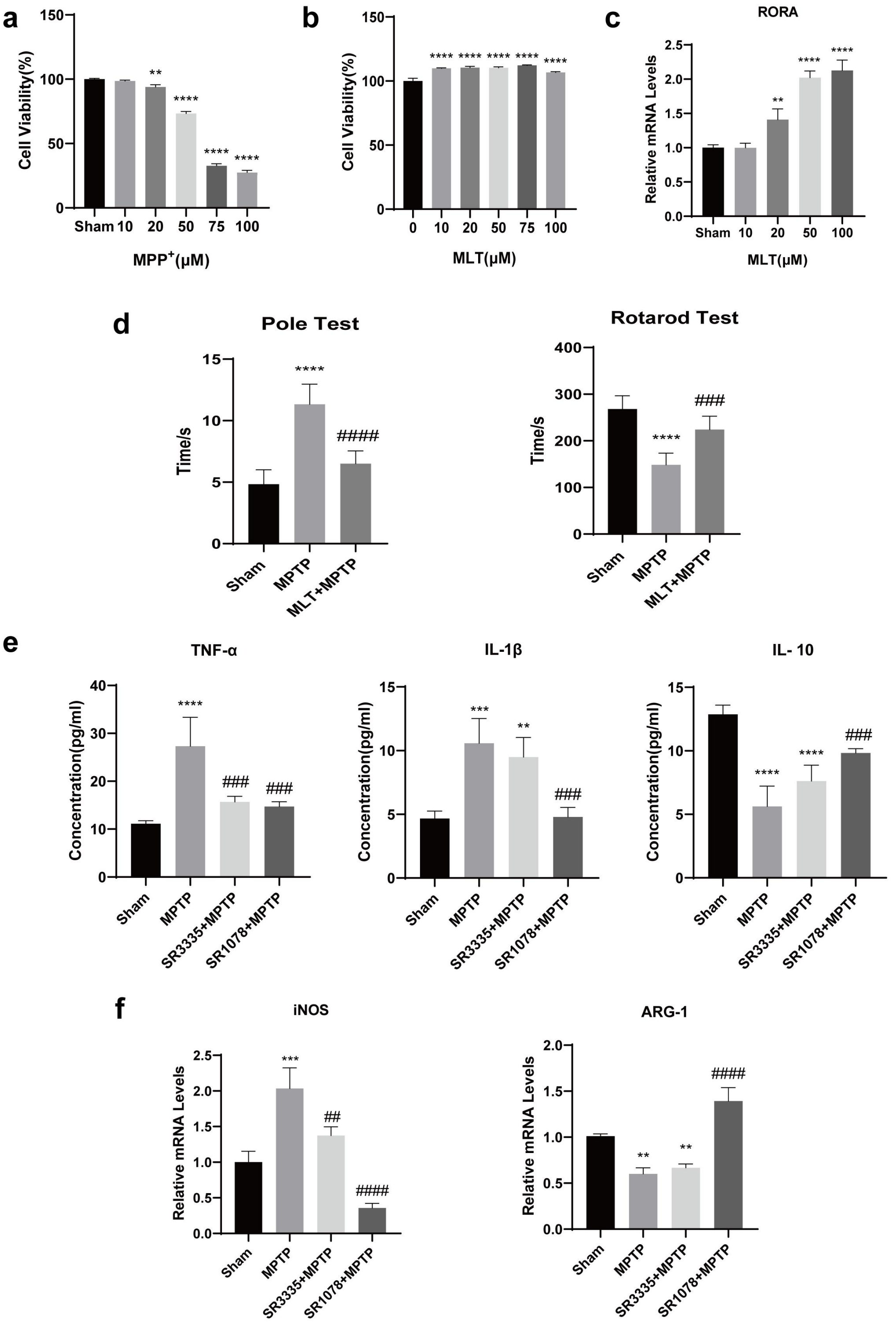

a
Cell Viability(%)
MPP+(μM)
Sham 10 20 50 75 100
b
Cell Viability(%)
MLT(μM)
0 10 20 50 75 100
c
RORA
Relative mRNA Levels
MLT(μM)
Sham 10 20 50 100
d
Pole Test
Rotarod Test
Time/s
Sham MPTP MLT+MPTP
e
TNF-α
IL-1β
IL- 10
Concentration(pg/ml)
Sham MPTP SR3335+MPTP SR1078+MPTP
f
iNOS
ARG-1
Relative mRNA Levels
Sham MPTP SR3335+MPTP SR1078+MPTP

Supplementary Figure 1

BV2 cells were treated with MPP^+ or MLT in different concentrations (0, 10, 20, 50, 75, 100 μM). The mice in MLT/SR3335/SR1078 group were pre-treated with MLT (20mg/kg, i.p.), SR3335 (15mg/kg, i.p.) and SR1078 (10mg/kg, i.p.) half an hour before MPTP (25mg/kg, i.p.) injection. MPTP, MLT, SR3335, SR1078 or equivalent saline were treated for 7 days.

(a,b) CCK-8 results showed the cell viability of BV2 cells in different groups. (c) The expressions of RORα after MLT treatment measured by RT-qPCR. (d) Motor functions of the control and MPTP groups were measured by pole and rotarod tests. (e) ELISA results indicated serum levels of TNF-α, IL-1β and IL-10 in different groups. (f) RT-qPCR showed the expressions of polarization markers iNOS and ARG-1 in midbrain tissue in different groups. Data are mean ± SD. (n=3 independent experiments. *, #, P<.05; **, ##, P<.01; ***, ###, P<.001; ****, ####, P<.0001; *, vs Sham group; #, vs MPTP group)

Figure 1b

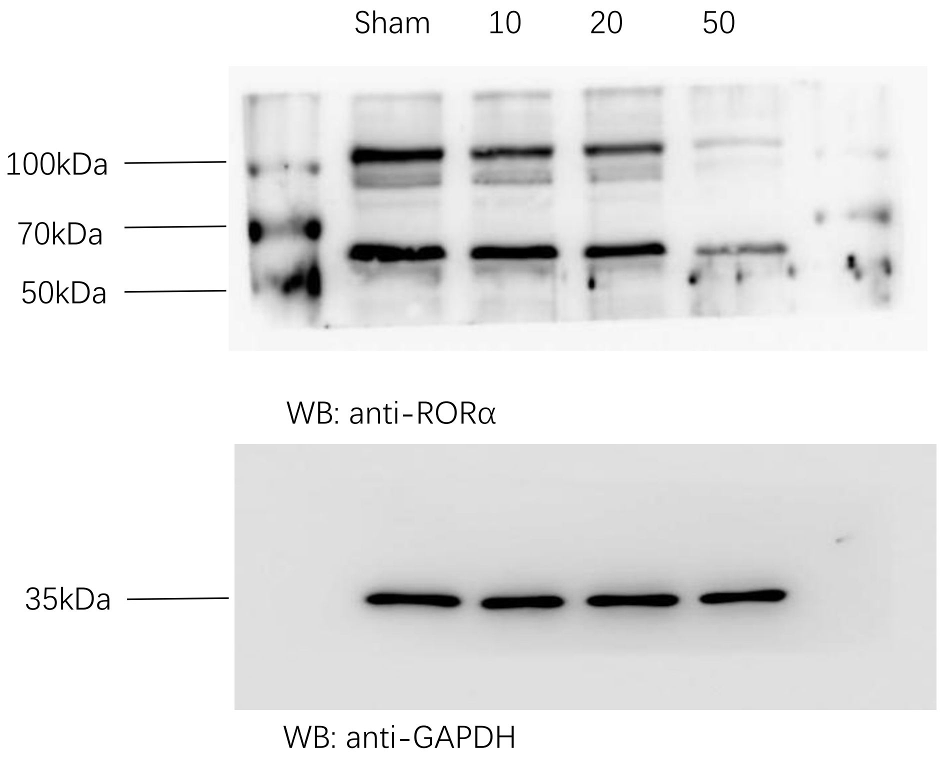

Figure 2b

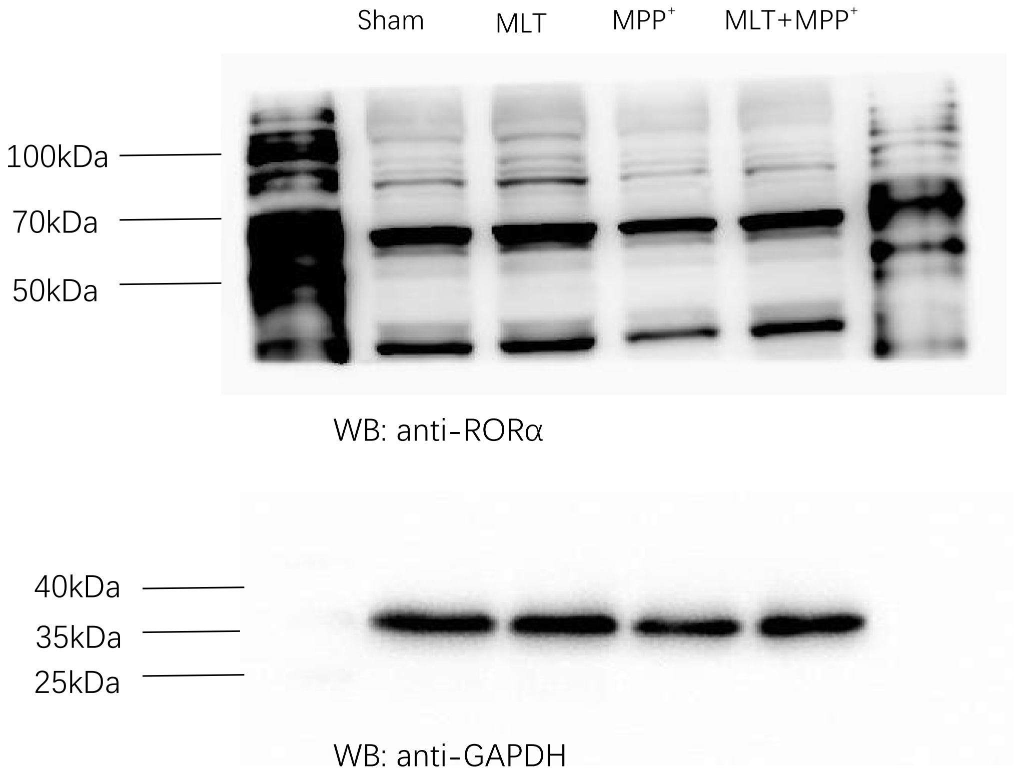

Figure 3b

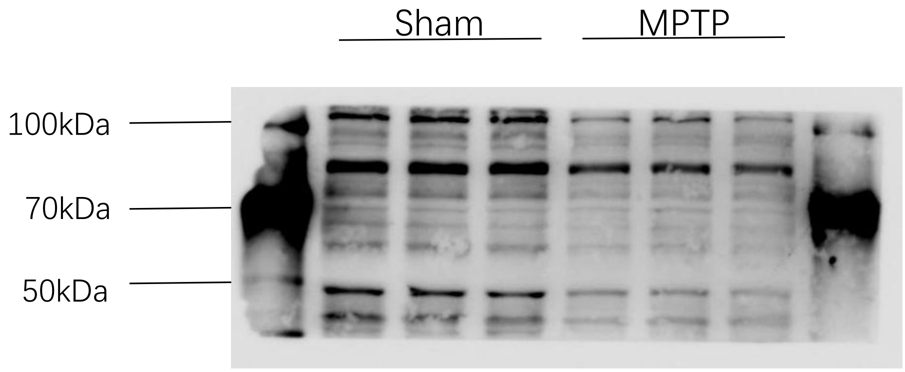

WB: anti-RORα

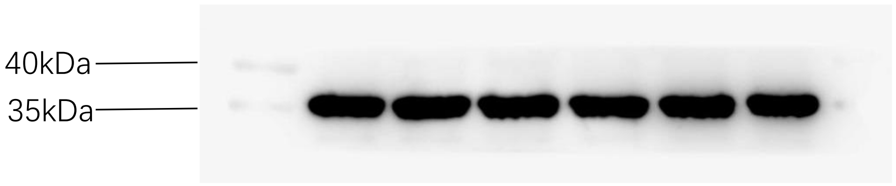

WB: anti-GAPDH

Figure 4b

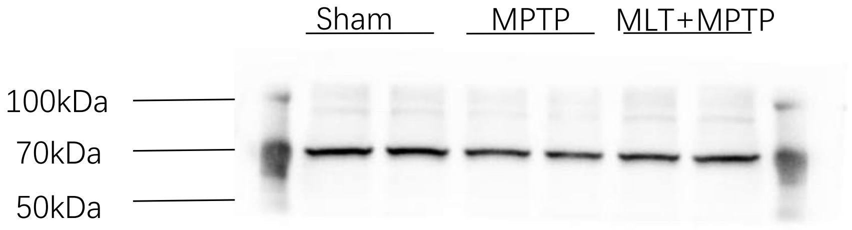

WB: anti-RORα

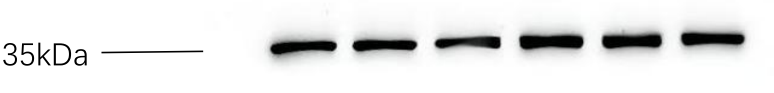

WB: anti-GAPDH

Figure 6a

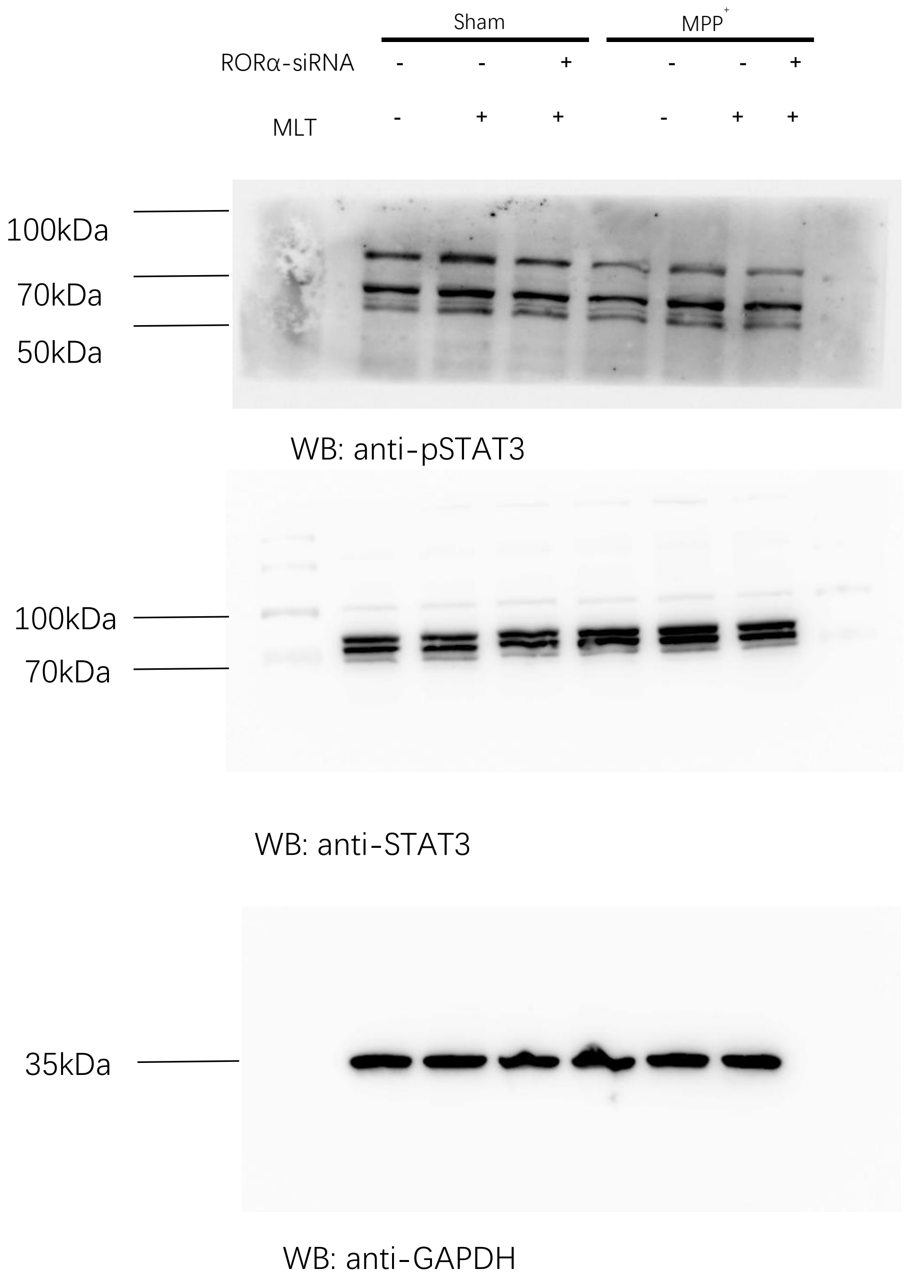
Sham
MPP+
RORα-siRNA - - + - - +
MLT - + + - + +
100kDa
70kDa
50kDa
WB: anti-pSTAT3
100kDa
70kDa
WB: anti-STAT3
35kDa
WB: anti-GAPDH

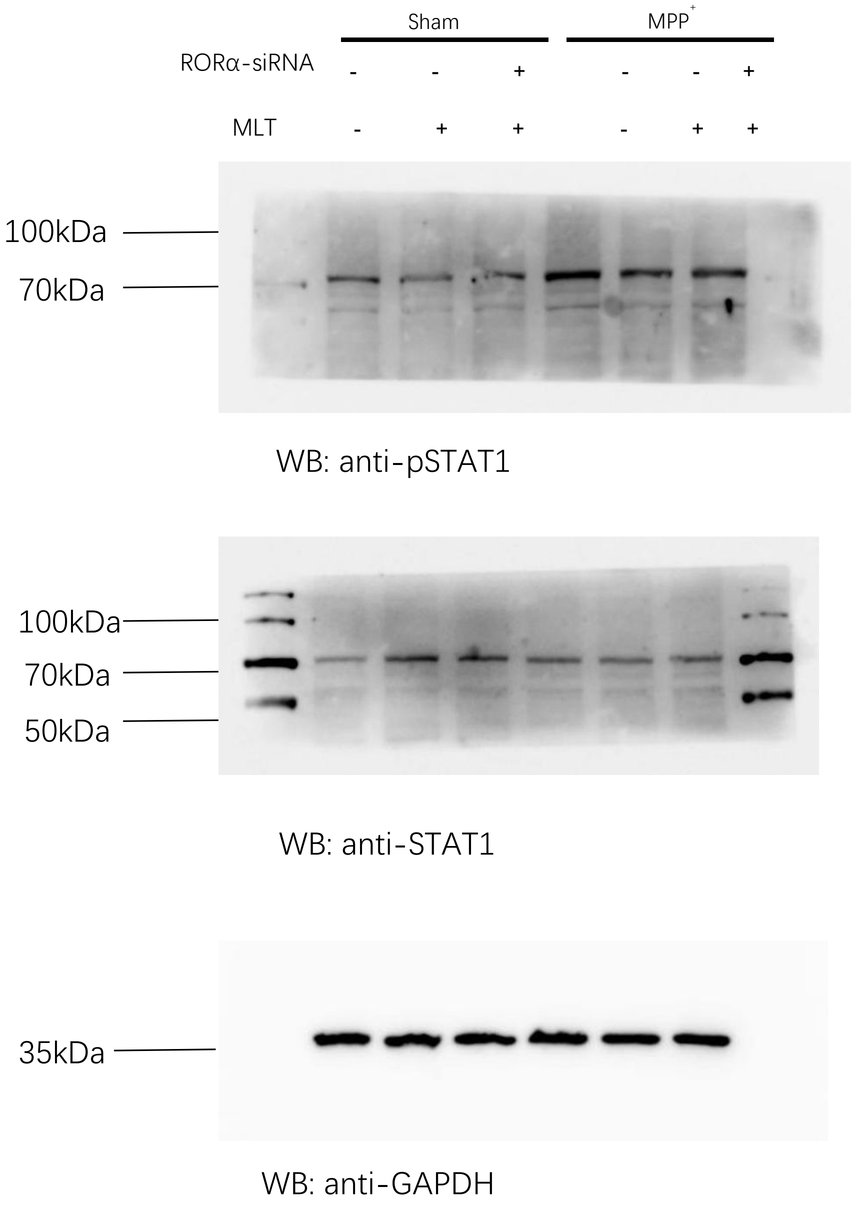
Sham
MPP+
RORα-siRNA - - + - - +
MLT - + + - + +
100kDa
70kDa
WB: anti-pSTAT1
100kDa
70kDa
50kDa
WB: anti-STAT1
35kDa
WB: anti-GAPDH
